# Comparative Transcriptome Profiling of Young and Old Brown Adipose Tissue Thermogenesis

**DOI:** 10.3390/ijms222313143

**Published:** 2021-12-05

**Authors:** Yumin Kim, Baeki E. Kang, Dongryeol Ryu, So Won Oh, Chang-Myung Oh

**Affiliations:** 1 Department of Biomedical Science and Engineering, Gwangju Institute of Science and Technology, Gwangju 61005, Korea; dbals123@gm.gist.ac.kr; 2 Department of Molecular Cell Biology, Sungkyunkwan University (SKKU) School of Medicine, Suwon 16419, Korea; baekikang@gmail.com (B.E.K.); freefall@skku.edu (D.R.); 3 Department of Nuclear Medicine, Seoul National University Boramae Medical Center, Seoul 03080, Korea

**Keywords:** brown adipose tissue, transcriptome, cold exposure, aging

## Abstract

Brown adipose tissue (BAT) is a major site for uncoupling protein 1 (UCP1)-mediated non-shivering thermogenesis. BAT dissipates energy via heat generation to maintain the optimal body temperature and increases energy expenditure. These energetic processes in BAT use large amounts of glucose and fatty acid. Therefore, the thermogenesis of BAT may be harnessed to treat obesity and related diseases. In mice and humans, BAT levels decrease with aging, and the underlying mechanism is elusive. Here, we compared the transcriptomic profiles of both young and aged BAT in response to thermogenic stimuli. The profiles were extracted from the GEO database. Intriguingly, aging does not cause transcriptional changes in thermogenic genes but upregulates several pathways related to the immune response and downregulates metabolic pathways. Acute severe CE upregulates several pathways related to protein folding. Chronic mild CE upregulates metabolic pathways, especially related to carbohydrate metabolism. Our findings provide a better understanding of the effects of aging and metabolic responses to thermogenic stimuli in BAT at the transcriptome level.

## 1. Introduction 

Brown adipose tissue (BAT) is a specialized site for uncoupling protein 1 (UCP1)-mediated non-shivering thermogenesis [1]. BAT dissipates chemical energy via heat generation to maintain the optimal body temperature against cold exposure and increases energy expenditure in response to excessive feeding [2]. Recent technical advances in the field of energy metabolism have revealed that the thermogenesis in BAT uses large amounts of intracellular triglycerides and glucose as the energy source [3,4], and thus activating the thermogenesis of BAT is a promising target for the treatment of obesity and related diseases, such as diabetes, dyslipidemia, and cardiovascular diseases [1,5]. Several human studies reported that BAT activities were affected by weather and climate [6,7,8]. There were strong associations between weather and BAT activity [6,8], and the Inuit, who live in the Arctic region, have genetic variants related to heat generation in BAT [7]. 

The amount of BAT and its thermogenic activity decrease with aging in both mice and humans [9]. Brown-like adipocytes in white adipose tissue (WAT), as well as brown adipocytes in BAT lose their thermogenic characteristics with aging [10]. Imaging studies using 18-fluorodeoxyglucose (18F-FDG) positron emission tomography (PET-CT) have revealed that young people have a higher stimulated/non-stimulated BAT ratio in the cervical-supraclavicular region than aged people [11]. Interscapular BAT abundantly exists in children aged <10 years but is dispersed in adults [12]. In humans, >90% metabolically active BAT is lost in their 50s and 60s [13]. 

Several approaches, such as cold exposure (CE), exercise, and beta 3-adrenergic receptor (β3-AR) agonists have been tried to activate the thermogenic activities of BAT [14,15,16,17,18]. Although their sample sizes were small, β3-AR stimulation has shown clinical benefits in clinical trials. Through acute administration of the mirabegron, the β3-AR agonist activated BAT metabolic activity and white adipose tissue (WAT) lipolysis in humans [16]. Chronic mirabegron therapy increased BAT activity and improved glucose homeostasis in both healthy and obese humans [17,18].

Although some human and animal studies have reported the metabolic benefits of activating BAT through these approaches [8,14,17,18], most clinical trials have been performed on young adults (ClinicalTrials.gov: NCT03793127, NCT03049462, and NCT02236962). In aged people, thermogenic stimuli do not activate the thermogenic activities of BAT [13]. Takeshi et al. identified cold-activated BAT by using PET-CT and 162 adult healthy volunteers aged 20–73 years [13]. In the same study, the incidence of activated BAT after 2 h of exposure to 19 °C was found to be 53% (44/83), 12.5% (1/8), and 0% (0/7) in humans during their 20s, 50s, and 60s, respectively. The underlying mechanism of this decline has not yet been elucidated.

Most chronic metabolic diseases, such as type-2 diabetes, develop with age [19]. Thus, the decline in the thermogenic activity and response to thermogenic stimuli with age is the major hurdle in harnessing BAT thermogenesis as a novel therapeutic strategy against metabolic disease. In this study, we compared the transcriptomic profiles of the BAT in both young and old mice in response to CE to find the molecular mechanisms underlying age-related dysfunctions in BAT. In addition, we compared the transcriptomic changes in BAT response to thermogenic stimuli such as acute severe CE (ACE), chronic mild CE (CCE), high-fat diet (HFD), and β3-adrenergic receptor (β3-AR) agonist *CL316243* treatment in young and old mice to determine which thermogenic stimulus is better. 

## 2. Results 

### 2.1. Comparison of BAT Transcriptome Profiles in Aging and Adaptive Thermogenesis

**Figure 1:**
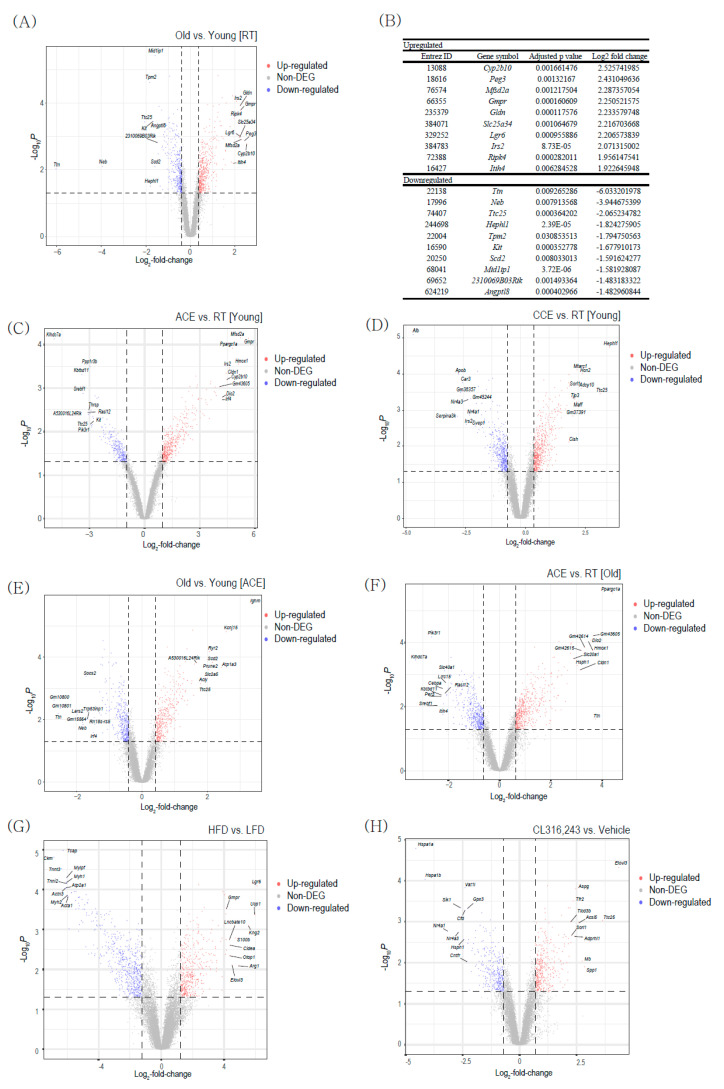
Differentially expressed genes (DEGs) between young and old brown adipose tissues (BAT). (**A**) Volcano plot of the DEGs between old vs. young BAT (GSE135391). (**B**) The list of top 10 upregulated and downregulated DEGs between old vs. young BAT. (**C**) Volcano plot of the DEGs between acute severe cold exposure (ACE) vs. room temperature (RT) in young BAT (GSE135391). (**D**) Volcano plot of the DEGs between chronic mild cold exposure (ACE) vs. room temperature (RT) in young BAT (GSE172021). (**E**) Volcano plot of the DEGs between old vs. young BAT upon ACE (GSE135391). (**F**) Volcano plot of the DEGs between ACE vs. RT in old BAT (GSE135391). (**G**) Volcano plot of the DEGs between high-fat diet (HFD) vs. low-fat diet (LFD) in young BAT (GSE112740). (**H**) Volcano plot of the DEGs between CL316243 treatment vs. vehicle treatment in young BAT (GSE98132).

The gene expression profile of BAT was analyzed using transcriptome datasets of ACE studies (GSE135391), CCE study (GSE172021), HFD induced thermogenesis study (GSE112740), and β3-AR stimulation (GSE98132). First, we analyzed the differentially expressed genes (DEGs) between young and aged BAT at room temperature (RT). We identified 438 upregulated and 366 downregulated genes (Adjusted *p*-value < 0.05, Figure 1A). Figure 1B shows the top 10 upregulated and top 10 downregulated genes ranked by fold change. *Cyp2b10* (cytochrome P450 2B10), *Peg3* (paternally expressed gene 3), and *Mfsd2a* (major facilitator superfamily domain-containing 2A) are highly upregulated in aged BAT compared with the levels in young BAT. *Ttn* (titin), *Neb* (nebulin), and *Ttc25* (tetratricopeptide repeat protein 25) genes are mostly downregulated in aged BAT compared with the levels in young BAT. The *Cyp2b10, Peg3, Mfsd2a*, and *Ttn* genes have previously been reported to play critical roles in adipocyte identity and metabolism [20,21,22,23]. However, the *Neb* and *Ttc25* genes are novel genes associated with BAT aging. Further studies are needed to investigate the role of these two genes in BAT. 

In young BAT, ACE (4 °C for 24 h) upregulated 444 genes and downregulated 266 genes (Figure 1C). CCE (gradual decrease from 23 °C to 10 °C, then 2 weeks of exposure) upregulated 514 genes and downregulated 369 genes (Figure 1D). Among the 444 upregulated genes upon ACE, only 33 are also upregulated upon CCE. Among the 266 downregulated genes upon ACE, only 25 are also downregulated upon CCE (Supplementary Figure S1). These small numbers of common genes suggest that each stress might trigger quite different signal responses for activating the BAT activity. 

A total of 788 genes were found to be differentially expressed, with 408 upregulated and 380 downregulated genes, between young and aged BAT upon ACE (Figure 1E). In old mouse BAT, ACE was found to upregulate 510 genes and downregulate 437 genes (Figure 1F). When we compared these DEGs with those between young and aged BAT at RT, 13 downregulated genes in aged BAT compared with young BAT at RT were found to be upregulated in aged BAT upon ACE. Additionally, 29 upregulated genes in old BAT compared with young BAT at RT were found to be downregulated in aged BAT upon ACE (Supplementary Figure S2). 

A total of 948 genes were found to be differentially expressed, with 399 upregulated and 549 downregulated genes, between HFD and low-fat diet in young BAT (Figure 1G). The thermogenic genes such as *Ucp1, Cidea,* and *Elovl3* were upregulated after HFD feeding in young BAT (Figure 1G). After β3-AR treatment, 381 genes were upregulated, and 326 genes were downregulated (Figure 1H). β3-AR treatment also increased the thermogenic gene *Elovl3* (Figure 1H).

### 2.2. Pathway Alterations in BAT

**Figure 2:**
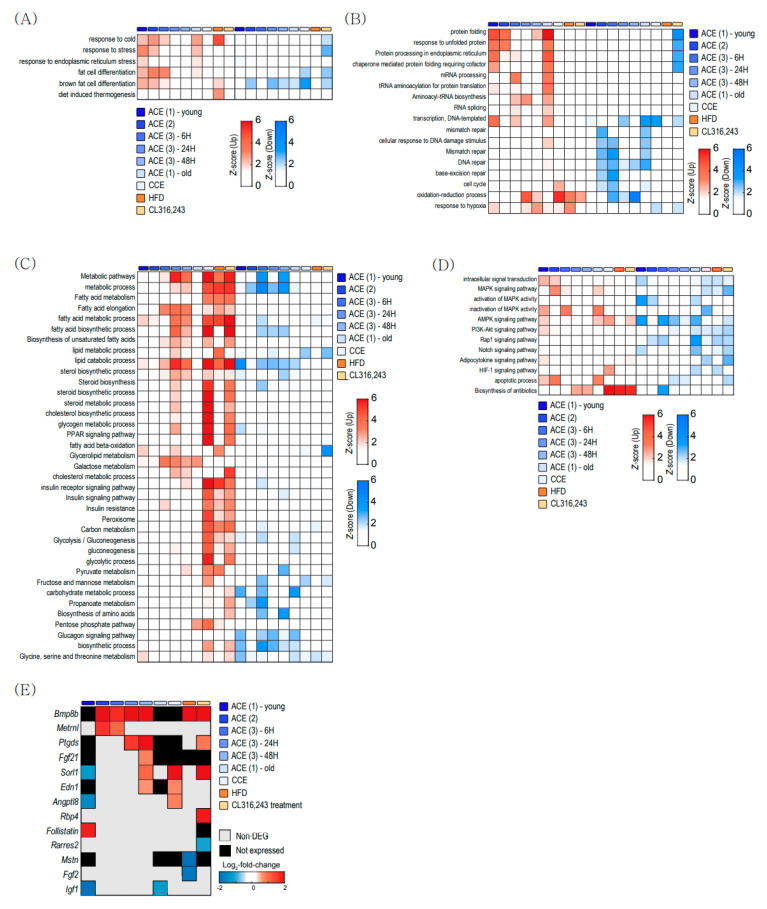
Clustered heatmap of functional enrichment analysis. (**A**) Pathways related to brown fat activity and stress. (**B**) Pathways related to protein processing. (**C**) Pathways related to metabolism. (**D**) Pathways related to signaling. (**E**) Genes related to adipokine secretion in brown adipose tissue. ACE, acute severe cold exposure; CCE, chronic mild cold exposure; HFD, high-fat diet.

We aimed to determine the biological characteristics of the DEGs in BAT that are associated with aging and/or other thermogenic stimuli. Thus, we performed gene ontology (GO) and Kyoto Encyclopedia of Genes and Genomes (KEGG) pathway analysis (Figure 2) using three ACE (GSE135391, GSE86590, and GSE119452), CCE (GSE172021), HFD (GSE112740), and β3-AR stimulation (GSE98132) transcriptomes.

ACE activates several pathways related to ‘response to cold’, ‘response to stress’, and ‘brown fat cell differentiation’ (Figure 2A). Both ACE and CCE did not induce significant changes in diet-induced thermogenesis (Figure 2A). ACE also activated endoplasmic reticulum (ER)-related protein folding and pathways related to the unfolded protein response in both young and old BAT (Figure 2B). This observation suggests that ACE increases the protein quality control related to cellular stress, and thus ACE cannot be a good candidate therapeutic strategy against the metabolic dysfunctions associated with aging. Figure 2C showed pathway changes related to metabolism. Metabolic pathways related to ‘fatty acid metabolism’, ‘cholesterol metabolic process’, and ‘insulin signaling pathway’ were upregulated in CCE, HFD, and CL316243 treatment. Figure 2D showed changes in signaling pathways. ACE upregulates ‘apoptotic process’ and ‘MAPK signaling pathway’.

BAT also plays a role as an endocrine organ that controls whole-body glucose and lipid metabolism by secreting adipokines, which are called ‘batokines’ [24]. To assess batokine secretion by thermogenic stimulation, we analyzed the expressions of ‘batokine’ genes (Figure 2E). Bone morphogenetic protein 8B (*BMB8B*) gene was increased after ACE, CCE, HFD, and β3-AR agonist stimulation. Fibroblast growth factor 21 (*FGF21)* was increased only 48 h after acute CE in young BAT. In old BAT, most genes did not show significant changes after thermogenic stimulation (Figure 2E).

### 2.3. Mitochondrial Gene Expression in Brown Adipose 

**Figure 3:**
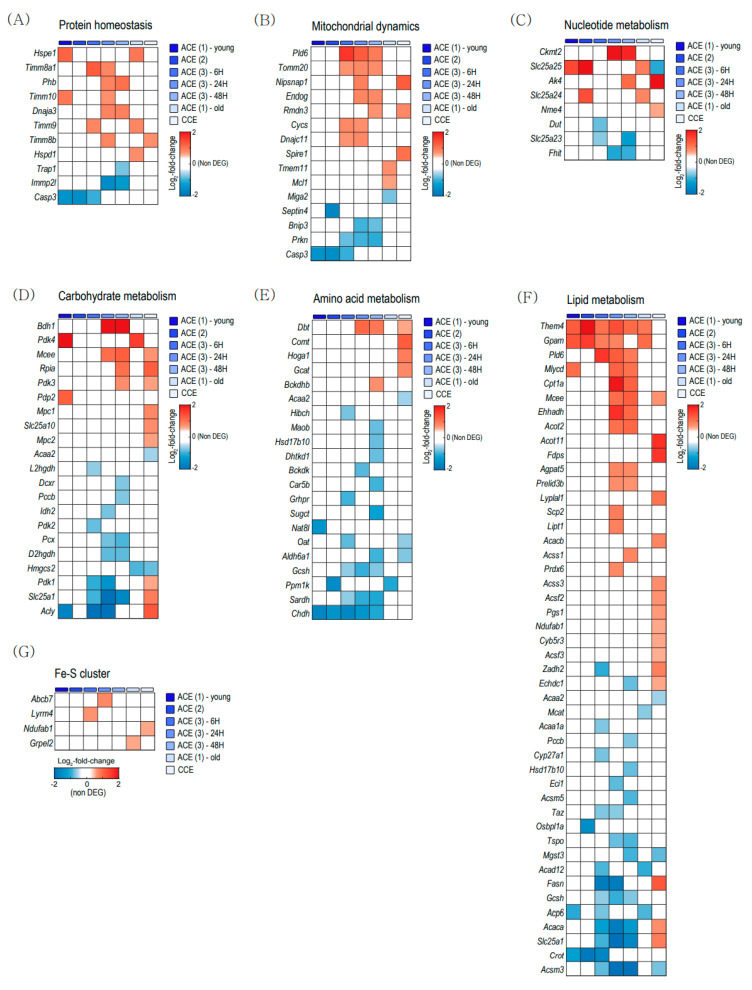
Heatmap visualization of differentially expressed mitochondrial genes (DEMGs) in brown adipose tissue (BAT). (**A**) DEMGs related to protein homeostasis. (**B**) DEMGs related to mitochondrial dynamics. (**C**) DEMGs related to nucleotide metabolism. (**D**) DEMGs related to carbohydrate metabolism. (**E**) DEMGs related to amino acid metabolism. (**F**) DEMGs related to lipid metabolism. (**G**) DEMGs related to the Fe-S cluster. ACE, acute severe cold exposure; CCE, chronic mild cold exposure.

To evaluate the mitochondrial changes with aging and CE in BAT, we analyzed the expressions of genes related to mitochondrial proteome in the Mitocarta 3.0 gene list [25]. Figure 3 shows heatmaps composed of the DEGs related to mitochondria in BAT. The rows of each heatmap represent mitochondrion-related genes with significantly changed expression levels based on fold change, and the columns are the comparative result of each group. 

Figure 3A showed DEGs related to protein homeostasis and Figure 3B showed the DEGs related to mitochondrial dynamics. Figure 3C showed the DEGs related to nucleotide metabolism. Both acute CE and chronic CE induces various changes in the expressions of genes related to mitochondrial proteostasis, dynamics, and nucleotide metabolism. 

Figure 3D–F shows DEGs related nutrients metabolism. Chronic CE upregulated genes related to carbohydrate metabolism and amino acid metabolism (Figure 3D,E). Lipid metabolism-related genes were upregulated in both acute CE and chronic CE (Figure 3F). Thioesterase superfamily member 4 (*Them4*) and glycerol-3-phosphate acyltransferase (*Gpam*) were commonly increased in the acute CE dataset (Figure 3F). Regarding the Fe-S cluster, only a few genes were differentially expressed in both acute CE and chronic CE (Figure 3G). 

### 2.4. Cold-Induced Changes in BAT

**Figure 4:**
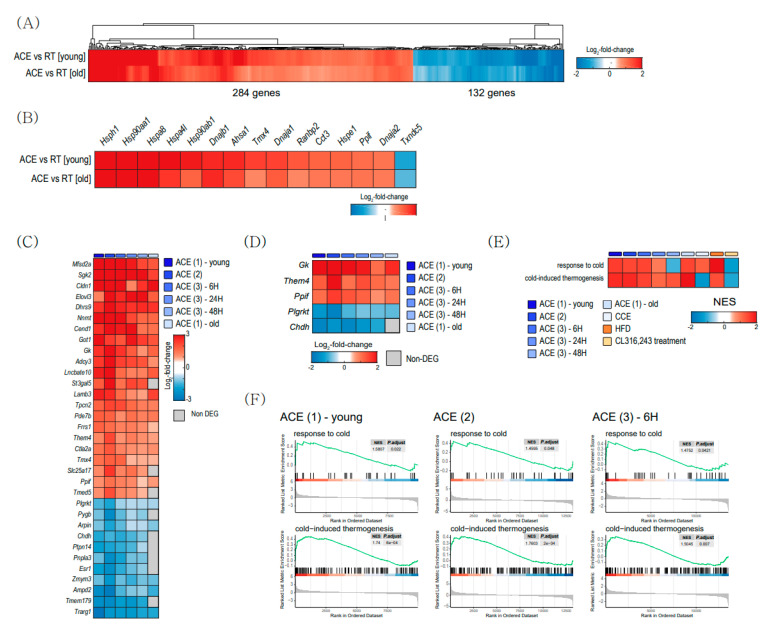
Cold-exposure-induced changes in gene expression in brown adipose tissue (BAT). (**A**) Heatmap of the common differentially expressed genes (DEGs) between acute severe cold exposure (ACE) vs. room temperature (RT) in young BAT, and DEGs between in old BAT (GSE135391). (**B**) Heatmap of the common DEGs related to protein folding pathway between ACE vs. RT in young BAT and DEGs between ACE vs. RT in aged BAT. (**C**) Common DEGs after CE in both young and old BAT from 3 datasets (GSE13591, GSE86590, and GSE119452). (**D**) Common mitochondrial DEGs from 4 datasets. (**E**,**F**) Gene set enrichment analysis (GSEA) result using all datasets. Heatmap (**E**) and enrichment plots (**F**) plots related to ‘response to cold’ and ‘cold-induced thermogenesis.

To determine specific changes between young and aged BAT after CE, we compared DEGs between ACE and RT in young BAT with DEGs in old BAT (GSE13591) (Figure 4). A total of 284 genes are commonly upregulated, and 132 genes are commonly downregulated in both DEGs (Figure 4A). Figure 4B shows the top genes related to protein folding in both DEGs. Heat shock protein family H (*Hsp110*) Member 1 (*Hsph1*), heat shock protein 90 alpha family class A Member 1 (*Hsp90aa1*), and heat shock protein family A Member 8 (*Hspa8*) are the top three upregulated genes in common DEGs. 

To analyze the general features related to CE, we compared DEGs from the other two datasets, GSE86590 and GSE119452. Figure 4C,D shows common DEGs and mitochondrial DEGs, respectively. Three mitochondrial genes, glycerol kinase (*Gk*), *Them4,* and peptidylprolyl isomerase F (*Ppif*) gene were upregulated after CE in young and old BAT (Figure 4D). The functional enrichment analysis showed that pathways related to ‘response to cold’ and ‘cold-induced thermogenesis’ were upregulated in both ACE and CCE (Figure 4E,F). 

### 2.5. Chronic Mild Cold Exposure Changes Metabolic Pathways in Brown Adipose Tissue

**Figure 5:**
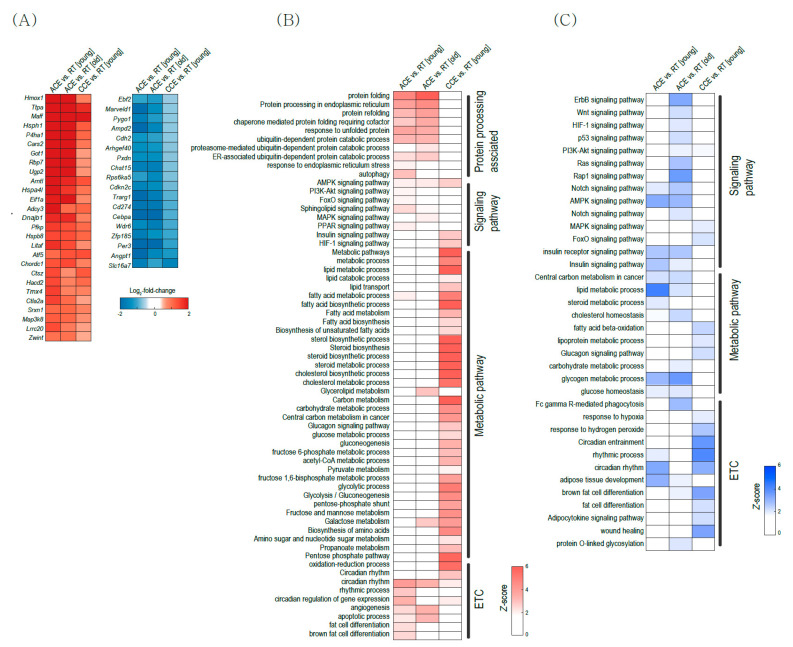
Transcriptional changes after chronic mild cold exposure (CCE) in brown adipose tissue (BAT). (**A**) Heatmap visualization of common differentially expressed genes (DEGs) between acute severe cold exposure (ACE) vs. room temperature (RT) in young BAT and DEGs between ACE vs. RT in aged BA and DEGs between CCE vs. RT in young BAT. (**B**,**C**) Gene Ontology Biologic Process (GOBP) and Kyoto Encyclopedia of Genes and Genomes (KEGG) pathway analysis result. Clustered heatmap of upregulated (**B**) and downregulated (**C**) pathways.

ACE and CCE have demonstrated beneficial effects in mice and humans by activating BAT thermogenesis [8,26,27]. Interestingly, DEG analysis (Figure 1) and pathway analysis (Figure 2) suggest that ACE and CCE use different signaling pathways for BAT activation. Thus, we next compared gene expressions and pathways between ACE and CCE using GSE135391 and GSE1127140 datasets (Figure 5). Figure 5A shows common genes in DEGs between ACE and RT in young BAT, DEGs between ACE and RT in old BAT, and DEGs between CCE and RT in young BAT. HSPs such as Hsph1, Hspa4l (heat shock protein family A (Hsp70) Member 4-like) and Hspb8 (heat shock protein family B (small) Member 8) are common upregulated DEGs (Figure 5A). Ebf2 (*early B-cell factor 2*) is a commonly downregulated DEG (Figure 5A). This gene is a specific marker gene for brown preadipocytes and drives brown adipocyte differentiation [28]. C/EBP*α* (CCAAT/enhancer-binding protein alpha) is also a commonly decreased DEG in CE, which triggers differentiation of white preadipocytes in mature white adipocytes [29].

Figure 5B shows common upregulated pathways and Figure 5C shows common downregulated pathways. ACE activates pathways related to protein processing, such as protein folding, protein ubiquitination, and unfolded protein response. However, CCE did not upregulate protein processing associated pathways and CCE upregulates metabolic pathways such as glucose and lipid metabolism. 

## 3. Discussion 

In this study, we analyzed transcriptomic profiles of BAT with aging and/or thermogenic stimuli such as CE, HTN, and β3-AR agonist. Old BAT showed decreased gene expression related to the lipid metabolism, such as stearoyl-CoA desaturase 2 (*Scd2*) and angiopoietin-like 8 (*Angptl8*), and increased gene expression related to obesity in WAT, such as *Peg3* [30]. Interestingly, 118 genes upregulated with aging are also upregulated in young BAT after ACE (Supplementary Figure S3). Even among the top ten upregulated genes in aged BAT compared with young BAT at RT (Figure 1B), three genes (*Mfsd2a*, GMP reductase (*GMPR*) [21], solute carrier family 25 member 34 (*Slc25a34*) [31]) are known as elevated genes related to CE in BAT. The upregulation of genes related to CE in old BAT might be a compensatory response to metabolic dysfunctions with aging, or this observation means that age-related stress might trigger a similar signaling pathway related to that induced by CE. 

The *Ttn* gene, which is downregulated in old BAT at RT, is one of the most downregulated genes in old BAT upon ACE (Figure 1B). This gene encodes a large protein called titin, which is an essential component of sarcomeres and plays an important role in muscle development [32]. BAT and the skeletal muscle arise from a common precursor (myf5-expressing precursor) cell. Accordingly, BAT and the skeletal muscle have been shown to share many genes as key regulators of their structures and functions [33,34]. 

Several studies have already reported the possible role of *Ttn* in adipose tissue. *Ttn* is significantly upregulated in the visceral adipose tissue of obese people, compared with the level in lean people [35]. Additionally, it is significantly downregulated in the BAT of obesity-prone rats, compared with the level in wild-type rats [36]. This finding suggests that *Ttn* might be a novel regulator of BAT. Further studies are needed to reveal the exact role of *Ttn* in the age-related changes of BAT. 

Intriguingly, thermogenic genes, such as uncoupling protein 1 (*Ucp1*), cell death-inducing DNA fragmentation factor alpha-like effector A (*Cidea*), cytochrome C oxidase subunit 8B (*Cox8b*), and ELOVL fatty acid elongase 3 (*Elovl3*), did not show significant differences in expression level between young and old BAT at RT. This observation suggests that decreased thermogenic activity with aging might result from the dysfunction of other core genes or the post-transcriptional changes of thermogenic genes in BAT. 

ACE significantly increased both *UCP1* and *PGC-1α* (Supplementary Figure S4). Intriguingly, CCE did not upregulate *PGC-1α* and CL316,243 treatment did not increase both *UCP1* and *PGC-1α*. *Cidea* increased only in HFD-induced thermogenesis. These results suggest that each stimulation may activate BAT through different thermogenic pathways. 

The functional enrichment analysis revealed that CE increased several pathways related to response to cold and stress in both young and old BAT (Figure 2). Both ACE and CCE also induced many changes related to mitochondrial functions in young and old BAT (Figure 3). However, pathways related to metabolism were increased in young BAT but not old BAT (Figure 2C). In old BAT, ACE increased only one gene related to carbohydrate metabolism, no gene related to amino acid metabolism, and two genes related to lipid metabolism (Figure 3D–F). CCE showed more upregulated pathways related to the carbohydrate and amino acid metabolism than ACE in young BAT. These findings suggest CCE might be an effective therapeutic strategy for improving metabolic dysfunction in old BAT. 

Aging did not cause transcriptional changes in thermogenic genes. CE activates thermogenesis-related genes in both young and aged BAT (Figure 4A,F). This observation means that a decrease in thermogenic gene expression is not the underlying cause of the reduced thermogenesis in aged BAT. Recently, Kazuki et al. also reported that the age-related impairment of BAT thermogenesis is not significantly associated with thermogenic genes and suggested post-translational regulated mitochondrial impairment, especially related to Fe-S cluster formation as a new underlying mechanism of BAT dysfunction with aging [37]. Among Fe-S cluster formation-related genes, ACE increased GrpE-like 2 (*Grpel2*) expression in old BAT (Figure 3G), which is a redox-sensitive protein against oxidative stress [38].

Pathway analysis revealed that ACE activated protein-folding-related pathways in both young and old BAT (Figure 2 and Figure 5). *Hsph1, Hsp90aa1* and *Hspa8* are the top three commonly upregulated genes in young and old BAT after ACE (Figure 4B). *Hsph1* encodes a member of the heat shock protein 70 family of proteins and this protein is a known marker of both human and mouse brown adipocytes [39]. *Hsp90aa1* encodes heat shock protein 90α, which is the isoform of the molecular chaperone Hsp90 [40]. This protein plays a role in lipid metabolism [41]. *Hspa8* encodes a member of the heat shock protein 70 family, which interacts with negative charged phospholipids and acts as a membrane chaperone [42]. These increases might be the result of protective responses to cold-induced stress in BAT [43], or these HSPs may participate in the BAT metabolism directly, because HSPs are specific molecular chaperones that play various roles in metabolism [44] as well as protein quality control [45]. 

Interestingly, circadian-rhythm–related pathways are changed according to aging and CE (Figure 5). Recently, many studies have reported that the circadian rhythm regulates energy metabolism, and chronodisruption by chronic desynchronization of circadian rhythms has detrimental effects on adipose tissue function and differentiation [46,47]. Thus, our finding suggests that normalization of the circadian disruption can be an effective strategy to treat the BAT dysfunctions associated with aging. 

Our study has several limitations. First, we did not obtain the transcriptome profile of aged BAT upon CCE. Comparison of the metabolic effects of ACE and CCE in aged BAT may provide us with a better understanding of the reduced thermogenic response to CE in aged BAT. Second, we used only transcriptome data for the evaluation of metabolic changes related to aging and CE. Integrated approaches using transcriptome with proteomics and metabolomics are needed to understand the age-related changes in BAT and confirm our analysis. Third, we used publicly available transcriptome for our study. Our results need to be verified by other methods such as real-time PCR. Further validation studies are needed.

In conclusion, our findings provided a better understanding of the various effects of thermogenic stimuli on BAT. Metabolic pathways, especially carbohydrate metabolism-related pathways, are more upregulated in BAT under CCE than other stimuli. Thus, CCE might be a better strategy for increasing metabolic activities and improving glucose homeostasis than other activating strategies in BAT. Further studies are needed to investigate the role of CCE in old BAT.

## 4. Material and Methods 

### 4.1. RNA-Seq Analysis of NCBI Gene Omnibus (GEO) Datasets

**Figure 6:**
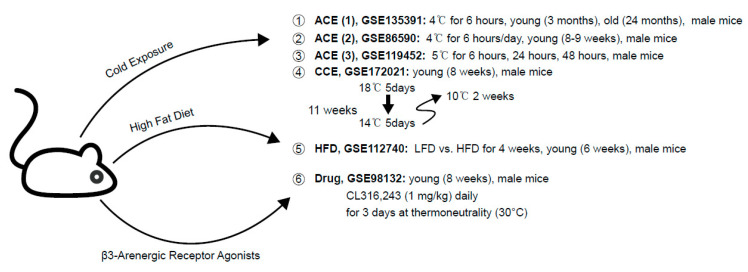
Information about transcriptome data that are used in this study.

Both RNA-seq data of ACE and CCE are deposited in GSE135391, GSE86590, GSE119452, and GSE172021. We used the GSE1127440 dataset for HFD-induced thermogenesis in BAT and GSE98133 dataset for β3-AR agonist-stimulated thermogenesis (Figure 6).

### 4.2. Identification of DEGs

Raw data were processed to calculate counts per million (cpm) through ‘edgeR’ [48] package in R software package (version 4.0.0 for Windows; http://cran.r-project.org, accessed on 11 October 2021), and those were converted to log_2_ scale followed by normalization using quantile normalization. Then, to identify DEGs from several datasets, we applied the integrative statistical method to the normalized log_2_-cpm. For each gene, to calculate the observed *T* value and log_2_-median-ratio between two conditions, Student’s *t*-test and log_2_-median-ratio were conducted, respectively. Then, random sampling 1000 times was performed to generate empirical null distributions for *T* value and log_2_-median-ratio. 

To generate the overall *p*-value for each gene, we calculate adjusted *p*-values by applying a two-tailed test for the measured *T* value and log_2_-median-ratio through their corresponding empirical distributions. Then, adjusted *p* values were combined into an overall *p*-value using Stouffer’s method [49]. For each comparison, we selected DEGs through two criteria: its overall *p*-value < 0.05 and the absolute log_2_-median-ratio > the mean of 2.5th and 97.5th percentiles of the empirical distribution for the log_2_-median-ratio (Supplementary Table S1). 

Venn diagrams which showed the comparison of DEGs between datasets were made through Oliveros, J.C. (2007–2015) Venny, an interactive tool for comparing listed data with Venn diagrams at 20-08-2021 (https://bioinfogp.cnb.csic.es/tools/venny/index.html, accessed on 11 October 2021).

### 4.3. Pathway Analysis: Kyoto Encyclopeida of Genes and Genomes (KEGG) and Gene Ontology Biologic Process (GOBP) Analysis

We conducted the functional enrichment analysis of the DEGs by using DAVID software [50]. Our GOBPs, KEGGs were selected by applying two criteria: its *p*-value < 0.05 and count of genes > 3.

### 4.4. Gene Set Enrichment Analysis (GSEA)

To assess the enrichment in our DEGs upon cold exposure in young and aged, we used GSEA through “clusterProfiler” in R software (version 4.0) [51]. We conducted GSEA analysis with 10,000 permutations, minGSSize = 3, maxGSSize = 800 and pvaluecutoff = 0.05.

## Data Availability

Data analyzed in this study were a re-analysis of existing data, which are openly available at locations cited in the reference section.

## Supplementary Materials

The following are available online at https://www.mdpi.com/article/10.3390/ijms222313143/s1.
