# Supplementary material for: Comparative Transcriptome Profiling of Young and Old Brown Adipose Tissue Thermogenesis
Source: Int J Mol Sci. 2021 Dec 5;22(23):13143. doi: 10.3390/ijms222313143 (PMC8658479; doi:10.3390/ijms222313143)

Figure S1. Venn diagram and heatmap showing the overlap between two differentially expressed genes (DEGs): DEGs between acute severe cold exposure (ACE) vs. room temperature (RT) in young BAT and DEGs between chronic mild exposure (CCE) vs. RT in young BAT.

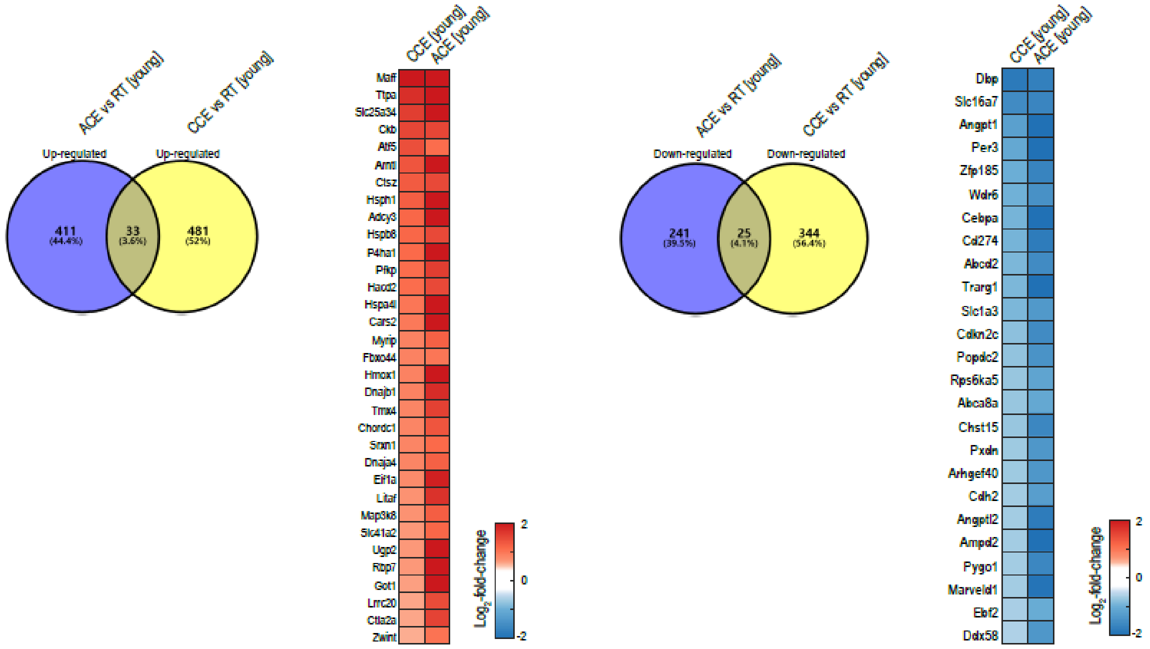

Figure S2. Venn diagram and heatmap showing the overlap between two differentially expressed genes (DEGs): DEGs between acute severe cold exposure (ACE) vs. room temperature (RT) in aged BA and DEGs between aged vs. young BAT at RT.

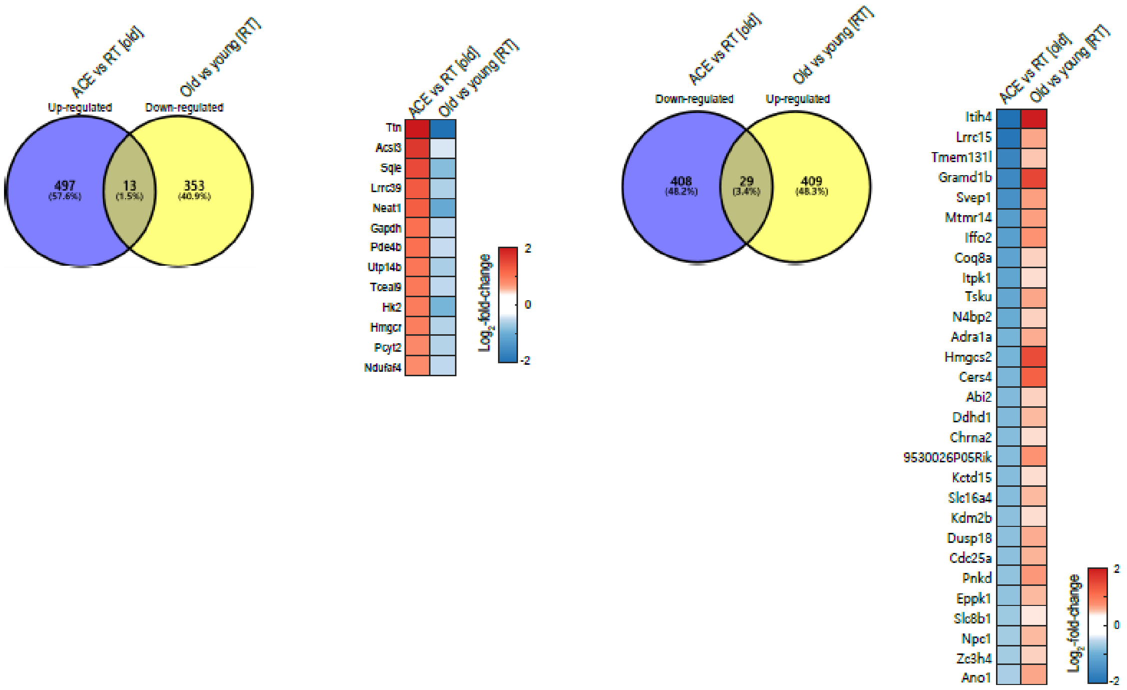

Figure S3. Venn diagram and heatmap showing the overlap between two differentially expressed genes (DEGs): DEGs between acute severe cold exposure (ACE) vs. room temperature (RT) in young BAT and DEGs between aged vs. young BAT at RT.

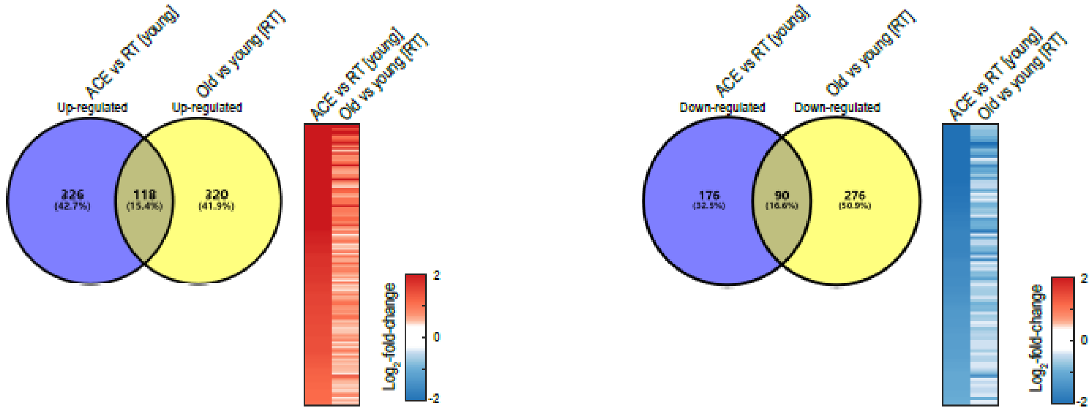

Figure S4. Thermogenic gene expression in each dataset.

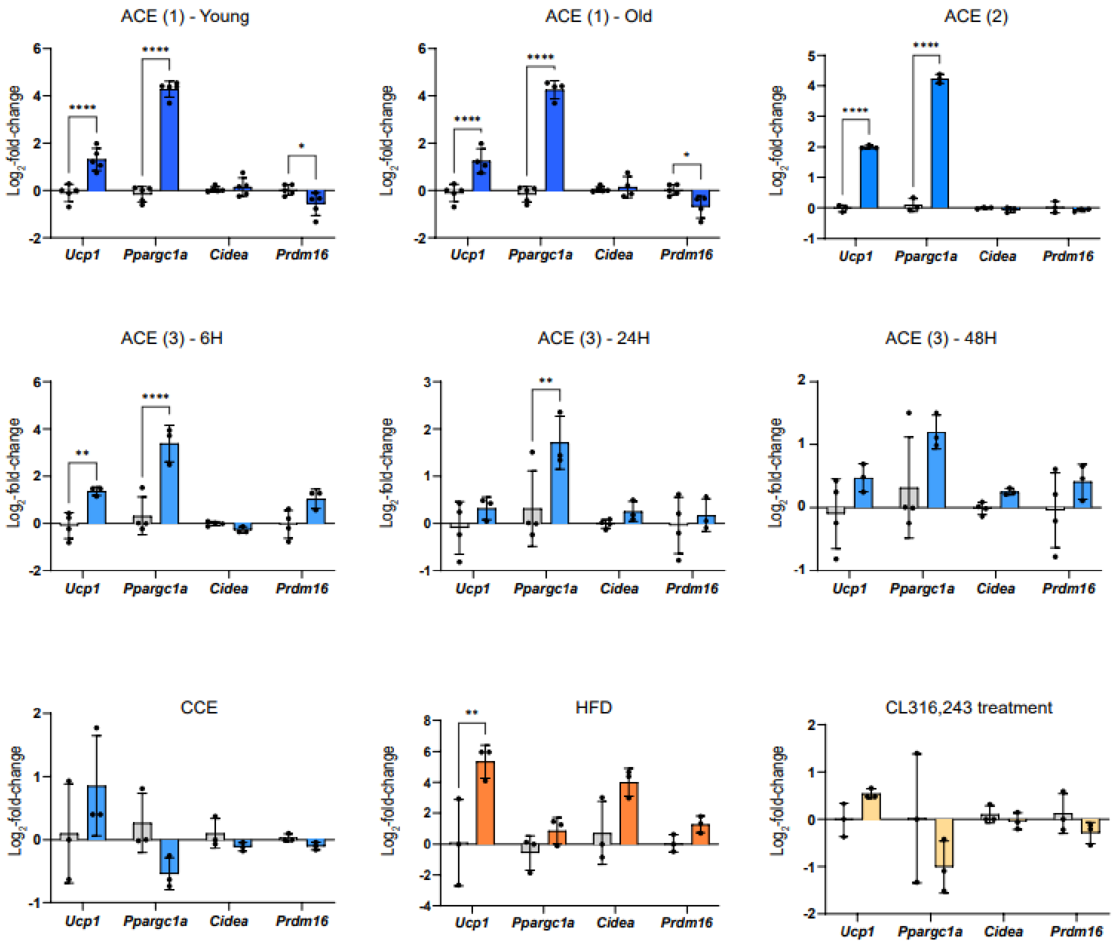

Supplement: Supplementary file 1 [file ijms-22-13143-s001.zip › ijms-1438666-Figures S1-S4.pdf]
